# Evaluation of a questionnaire to assess selected infectious diseases and their risk factors: Findings of a multicenter study

**DOI:** 10.1007/s00103-014-2052-y

**Published:** 2014-10-10

**Authors:** Claudia Sievers, Manas K. Akmatov, Lothar Kreienbrock, Katja Hille, Wolfgang Ahrens, Kathrin Günther, Dieter Flesch-Janys, Nadia Obi, Karin B. Michels, Julia Fricke, Karin H. Greiser, Rudolf Kaaks, Hans-Hartmut Peter, Frank Pessler, Alexandra Nieters, Gérard Krause

**Affiliations:** 1 Department of Epidemiology, Helmholtz Centre for Infection Research, Inhoffenstraße 7, 38124 Braunschweig, Germany; 2 Department of Biometry, Epidemiology and Information Processing, WHO Collaborating Centre for Research and Training in Veterinary Public Health, University of Veterinary Medicine Hannover, Hannover, Germany; 3 Leibniz Institute for Prevention Research and Epidemiology—BIPS, Bremen, Germany; 4 Institute for Statistics, Faculty of Mathematics and Computer Science, University of Bremen, Bremen, Germany; 5 Department of Cancer Epidemiology, Clinical Cancer Registry, University Cancer Center Hamburg (UCCH), Hamburg, Germany; 6 Institute for Prevention and Cancer Epidemiology, University Medical Center Freiburg, Freiburg, Germany; 7 Obstetrics and Gynecology Epidemiology Center, Department of Obstetrics and Gynecology, Brigham and Women’s Hospital, Harvard Medical School, Boston, MA USA; 8 Division of Cancer Epidemiology, German Cancer Research Center (DKFZ), Heidelberg, Germany; 9 Institute for Social Medicine, Epidemiology and Health Economics, Charité- Universitätsmedizin Berlin, Berlin, Germany; 10 Center for Chronic Immunodeficiency, University Medical Center Freiburg, Freiburg, Germany; 11 Institute for Experimental Infection Research, TWINCORE Center for Experimental and Clinical Infection Research, Hannover, Germany; 12 Helmholtz Centre for Infection Research, Braunschweig, Germany; 13 Hanover Medical School, Hannover, Germany

**Keywords:** Survey validity, Infections, Questionnaires, Self-report, German National Cohort (GNC), Infektionen, Fragebogen zu Infektionskrankheiten, Selbsteinschätzung, Validierung, Nationale Kohorte (NaKo)

## Abstract

**Background/objectives:**

The risk to die from an infectious disease in Germany has been continuously decreasing over the last century. Since infections are, however, not only causes of death but risk factors for diseases like cardiovascular diseases, it is essential to monitor and analyze their prevalence and frequency, especially in consideration of the increased life expectancy. To gain more knowledge about infectious diseases as risk factors and their implications on the condition and change of the immune status, the German National Cohort (GNC), a population-based prospective cohort study, will recruit 200,000 subjects between 2014 and 2017. In Pretest 1, a feasibility study for the GNC, we evaluated a self-administered and self-report questionnaire on infectious diseases and on the use of health care facilities (hereinafter called “ID Screen”) for feasibility and validity.

**Methods:**

From August–November 2011, 435 participants between the ages of 20–69 completed the ID Screen. All subjects had been recruited via a random sample from the local residents’ registration offices by 4 of the 18 participating study centers. The questionnaire encompasses 77 variables in six sections assessing items such as 12-month prevalence of infections, cumulative prevalence of infectious diseases, visit of health care facilities and vaccination. The feasibility was amongst others evaluated by assessing the completeness and comprehensiveness of the questionnaire. To assess the questionnaires ability to measure “immune status” and “susceptibility to infections”, multivariate analysis was used.

**Results:**

The overall practicability was good and most items were well understood, demonstrated by < 2/33 missing questions per questionnaire and only three variables: vaccination for influenza and pneumococci and infection with chickenpox had a frequency > 5 % of missing values. However, direct comparison of the items 12-month prevalence and lifetime prevalence of nephritis/pyelitis showed poor agreement and thereby poor understanding by 80 % of the participants, illustrating the necessity for a clear, lay person appropriate description of rare diseases to increase comprehensibility. The questionnaire will be used to support the assessment of immune dysfunction and frequency of infection. An analysis of these constructs in an exploratory factor analysis revealed limited applicability due to low interitem correlation (Cronbach’s α < 0.5). This is corroborated by the extraction of more than one factor with a Kaiser–Meyer–Olkin measure of 0.6 instead of a unidimensional latent construct for “immune status”.

**Conclusion:**

All in all, the ID Screen is a good and reliable tool to measure infectious diseases as risk factors and outcome in general, but requires a better translation of infection specific terms into lay person terms. For the assessment of the overall immune status, the tool has strong limitations. Vaccinations status should also rather be assessed based on vaccination certificates than on participants’ recall.

**Electronic supplementary material:**

The online version of this article (doi: 10.1007/s00103-014-2052-y) contains supplementary material, which is available to authorized users.

## Abstract (de)

**Einführung/Ziele:**

Infektionskrankheiten sind in Industrieländern nicht mehr die führenden Ursachen für Morbidität und Mortalität. Als Risikofaktoren für nichtübertragbare, wie z. B. kardiovaskuläre Krankheiten, spielen sie jedoch nach wie vor eine wichtige Rolle. In der Nationalen Kohorte (NaKo), einer prospektiven Kohortenstudie, die zwischen 2014 und 2017 in Deutschland den Gesundheitszustand von 200.000 Bürgern misst, ist die Erfassung von Infektionen und ihren Risikofaktoren eine hervorgehobene Komponente. Im Pretest 1 zur NaKo wurde ein Fragebogen, der u. a. Infektionen und medizinische Behandlungen mittels Selbstangabe und Selbsteinschätzung erfasst (ID-Screen), auf Machbarkeit und Vollständigkeit evaluiert.

**Methode:**

Im Zeitraum von August bis November 2011 füllten in 4 der 18 teilnehmenden Studienzentren 435 über die Einwohnermeldeämter ermittelte Probanden im Alter zwischen 20 und 69 den Fragebogen aus. Dieser bestand aus 77 Variablen in 6 Abschnitten und erfasste unter anderem 12-Monats- und Lebenszeitprävalenzen von Infektionen, Klinikbesuchen und Impfungen. Zur Evaluierung wurden die Ergebnisse auf Vollständigkeit und Verständnis überprüft. Zudem wurde mittels multivariater Analyse ermittelt, ob der Fragebogen wie geplant den Immunstatus und die Infektanfälligkeit erfasst.

**Ergebnis:**

Der Fragenbogen war gut verständlich und wurde von den Probanden akzeptiert, wie man der geringen Zahl an fehlenden Daten pro Fragebogen (< 2/33 Fragen) entnehmen konnte. Zudem hatten nur die 3 Fragen zu Impfungen gegen Influenza, Impfungen gegen Pneumokokken sowie zu Infektionen mit Windpocken mehr als 5 % fehlende Messwerte. Vergleicht man die Erfassung der 12-Monats-Prävalenz und der Lebenszeitprävalenz für Nieren-/Nierenbeckeninfektionen, so findet man bei 4 von den 5 betroffenen Probanden (80 %) eine mangelnde Übereinstimmung. Dies verdeutlicht, dass medizinische Begriffe und seltene Erkrankungen für Laien als Zielpersonen entsprechend erklärt werden müssen. Um die Eignung des ID-Screens zur Messung des Immunstatus und der Infektanfälligkeit zu bestimmen, wurde eine explorative Faktorenanalyse durchgeführt. Hier hat sich u. a. gezeigt, dass für das Konstrukt „Immunstatus“ eine zu geringe Inter-Item-Korrelation (Cronbach’s α < 0,5) vorliegt und mit einem KMO von 0,6 mehr als ein Faktor extrahiert wurde. Somit lässt sich der Immunstatus mit dem ID-Screen nicht als eine latente Variable abbilden.

**Schlussfolgerung:**

Der Fragebogen ist ein geeignetes Instrument, um Infektionskrankheiten zu erfassen. Für Laien als Zielgruppe sollten Fachbegriffe jedoch besser übersetzt werden. Um den Immunstatus als solchen zu erfassen, hat der ID-Screen starke Einschränkungen. Statt in einer erinnerungsbasierten Impfabfrage sollten Impfungen besser über die Informationen im Impfpass erfasst werden.

With the increased life expectancy in the German population, the proportion of deaths from leading causes such as cancer, coronary heart diseases and stroke will further increase [1, 2]. Numerous risk factors for the major disease-related causes of death are already known and have been characterized including smoking, alcohol, physical activity and nutrition. The role of bacterial and viral infections, chronic inflammatory processes or impairment of the immune system in the development of the diseases named above still have to be assessed.

Starting in 2014, the German National Cohort (GNC), a large nationwide prospective cohort study with an anticipated sample of 200,000 individuals between the ages of 20 and 69 years, will collect information on the current and former health status of the German population as well as store the individual’s biosamples such as blood, urine, stool and nasal swab [3, 4].

One of the distinguished aspects of the GNC compared to other national and international prospective cohort studies will be the assessment of infectious diseases. The Working Group Infection & Immunity of the GNC identified five topics (T) for which a cohort study within an aging population is an ideal and unique approach to be addressed. These are immune senescence/dysfunction and vaccines (T1), chronic viral infections (T2), respiratory infections (T3), bacterial infections (including resistance factors) and zoonoses (T4) and gastrointestinal infections (T5). To address T1, vaccination status and non-infectious risk factors have to be determined. T2–T5 are based on a determination of incidence and burden of infections, amongst others. To assess these questions a new infectious disease questionnaire (ID Screen) as complementary tool to a comprehensive medical standardized interview (core questionnaire) was developed to identify individuals at risk of immune dysfunction. Furthermore, it assesses frequent infections as well as exposure to pets and animal husbandry to identify potential sources of zoonotic diseases. Parts of the questionnaire are based on the immune system assessment questionnaire (ISAQ), developed at the Center for Chronic Immunodeficiency, University Medical Center Freiburg [5].

As the ID Screen was for the first time applied in Pretest 1, the first feasibility study of the GNC, an evaluation concerning its feasibility and validity is necessary. The ID Screens basic requirements were that it should (1) be self-administered and self-reported, (2) be short (less than 10 min to complete), (3) measure infections including resistance factors, (4) measure susceptibility to infections and immune status and (5) be reliable and valid.

To evaluate the feasibility and validity of the ID Screen as well as the potential for its application as a take-home questionnaire compared to an on-site questionnaire, four study centers participated in implementing the ID Screen.

## Methods

### Study design

The examination of subjects during Pretest 1 took place from August–November 2011; subjects between the ages of 20–69 years were recruited via random sampling through the regional registration offices. Due to oversampling of older participants, the envisaged distribution was 10 % for the age groups 20–29 and 30–39 and 26.7 % for the age groups 40–49, 50–59 and 60–69. Protocols for further recruitment procedures, like area-based recruitment, follow-up for nonresponders and incentives were developed independently in each study center.

Participants undertook a medical examination as well as the GNC core questionnaire assessing health and dietary aspects on-site. Four study centers, Bremen, Hamburg, Heidelberg and Freiburg additionally applied the ID Screen, with Bremen and Hamburg providing a take-home questionnaire to be picked up later by study center personnel and Heidelberg and Freiburg conducting an on-site questionnaire. Participation was limited to subjects with sufficient knowledge of the German language.

### ID Screen

The ID Screen was a self-report and self-administered questionnaire, divided into six sections (S): 12-month cumulative incidence of certain infections (S1), lifetime prevalence of certain infectious diseases (S2), utilization of health care facilities (S3), 12-month cumulative incidence of antibiotic intake (S4), vaccination status for influenza and pneumococcal infection (S5) and contact to pets (S6), encompassing in total 77 variables. Only the 33 nonfilter questions that could be answered independent of the prior answer being yes, were evaluated. See supplementary material for the complete translated questionnaire.

### Core questionnaire

The ID Screen is a complementary tool to the computer-assisted personal interviewing (CAPI), assessing the comprehensive medical state of the participants. The following 8 items of this core questionnaire were used in the evaluation of the ID Screen:

“Has a physician ever diagnosed [rheumatic diseases (1); autoimmune diseases (2); diseases of the skin (3); asthma (4); allergies (5): hay fever, bee venom, food, dust mites, animal hair, contact allergy, drug allergy; chickenpox (6); shingles (7); sepsis (8)]?” to be answered with “yes”, “no” or “don’t know”.

### Feasibility/validity

To assess the feasibility of the ID Screen, aspects concerning time for completion and compliance especially with regard to the different fill in scenarios (take-home vs. on-site) were evaluated. The compliance is reflected in the participation and the total number of missing values per participant for the 33 nonfilter questions, with an incomplete questionnaire being defined as more than 50 % of the questions containing missing values.

#### Internal validity

Aspects of internal validity were addressed by analyzing the frequency of missing, “don’t know” and “no answer” values for the 33 nonfilter questions; a frequency < 5 % was considered acceptable. Comments by participants were taken into consideration to evaluate comprehension of the ID Screen. Furthermore, a direct comparison of related questions, such as 12-month prevalence/lifetime infection and antibiotic prescriptions/use of health care facilities was used, to assess unsuitably phrased questions leading to a lack of understanding by the participants. To evaluate the usefulness of the categorical scale used for the items in section S1 and S4, the frequency with which each category was answered was measured.

#### Reliability

To assess the reliability, the agreement between answers for duplicate variables in the core questionnaire of the GNC and the ID Screen, Cohen’s κ was calculated. The proposed interpretation for strength of agreement is 0: poor; 0.01–0.2 slight; 0.21–0.40: fair; 0.41–0.60: moderate; 0.61–0.80: substantial and 0.81–1: almost perfect [6].

#### Construct validity

The ID Screen was designed as a tool to measure amongst others (1) susceptibility to infections and (2) immune status. Exploratory factor analysis (EFA) was used with the aim to create a score for each of the two constructs. The variables included in the construct “susceptibility”, all variables from section 1, were recoded to present the midpoints over the categories (none: 0; 1–2 times: 1.5; 3–4 times: 3.5; 5–6 times: 5.5 and more than 6 times: 7). For the construct “immune status” the following variables were clustered: allergies, rheumatic-, autoimmune-, skin diseases and asthma from the core questionnaire of the GNC as well as the variables: surgery, removal of 2^nd^ lymphoid organs, variables from section 1 (midpoints) and number of shingle episodes from the ID Screen. The variable “removal of the spleen” was removed from the analysis due to a conditional variance of zero. All non-ordinal data were recoded to dichotomous variables with no = 0 and yes = 1. “Don’t know” answers were recoded as missing data.

To show that there is covariation among the included variables, the Kaiser–Meyer–Olkin measure (KMO), where a minimum value of 0.5 is required, was used to check the sampling adequacy of the constructs [7]. Pearson correlation was used to examine the correlation r between the contributing variables [8]. The internal consistency was examined by Cronbach’s α which can be interpreted as follows: α ≥ 0.9: excellent; 0.7–0.9: good; 0.6–0.7: acceptable; 0.5–0.6: poor and α < 0.5: unacceptable [9]. Applying the scree test criterion resulted in the extraction of one factor for the construct “susceptibility” and four factors for the construct “immune status”. For better allocation of the items per factor and therefore better interpretation of the factors “immune status”, an orthogonal rotation (Varimax) was applied [8]. Only variables with a factor loading > |0.4| are considered for interpretation [8, 10].

All statistical analyses except the estimation of confidence limits were computed with SAS® 9.2. The confidence limits were calculated in EXCEL 2010 using an approximation based on Rothman [11].

## Results

### Feasibility

Between the four study centers, 467 subjects were recruited to participate in the medical examination and the comprehensive medical core questionnaire. A total of 435 (93 %) subjects participated in the ID Screen, with the age distribution between 18 and 70 years and a mean age of 47.4 ± 14.4 years.

General characteristics of the participants differentiated by study center are shown in Tab. 1. The participation rate for the take home questionnaire was 89 % and for the on-site questionnaire 98 %.

**Table 1:** Characteristics of subjects by participating study centers and analysis of compliance and time for completion

	Bremen *n* = 87	Hamburg *n* = 90	Heidelberg *n* = 105	Freiburg *n* = 156
Women (%)	57	57	46	51
Mean age + SD (years)	49.6 ± 13.6	46.8 ± 15.6	44.1 ± 14.9	48.9 ± 13.4
Migration background^a^ (%)	33.3	30.8	37.7	26.3
Questionnaire	Take home	Take home	Study center	Study center
Participation ID Screen (%)	87	90	95	99
Median time to complete (min)	10	10	6	8
[Inter quartile range]	[6–17]	[6–14.5]	[5–8]	[5–10]
Missing responses per questionnaire (mean)	2.0/33	0.7/33	0.5/33	0.6/33
CI_95 %_	1.5–2.5	1.0–1.4	0.8–1.1	0.8–1.1
Min–max	0–18	0–12	0–9	0–9
Incomplete questionnaires (> 16/33 missing values)	1	–	–	–
Subjects receiving help from study nurse (*n*)	Not applicable	Not applicable	1	4

^a^Migrations status was determined according to Schenk et al. [12]

The mean time to fill in the questionnaire as well as the proportion of missing responses per questionnaire was higher among the take-home than the on-site questionnaires. One of the take-home questionnaires was rated as incomplete due to 18 missing values.

### Internal validity

The aspect of internal validity to be assessed was the analysis of missing answers per variables to identify inaccurate items. Three variables of the categories vaccination and childhood diseases showed a frequency of missing values > 5 %, with vaccination against influenza being 12 %, against pneumococci 25 % and lifetime prevalence of chickenpox infection 12 %, respectively.

**Fig. 1:**
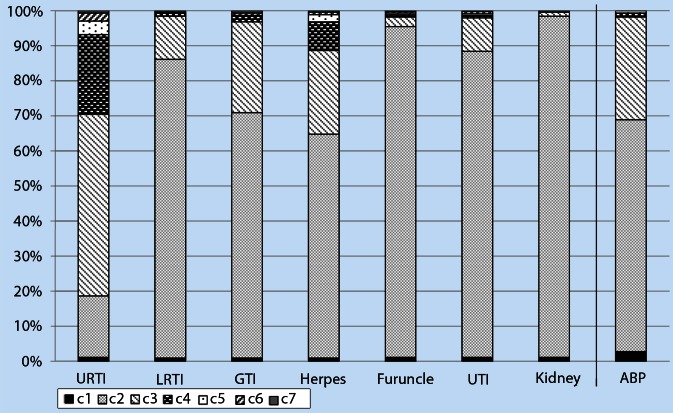
Separation of data points for 12-month prevalence of infections and antibiotic prescription across the categories. In section 1 of the ID Screen the 12-month prevalence of the infections: upper respiratory tract infections (*URTI*), lower respiratory tract infections (*LRTI*), gastrointestinal infections (*GTI*), infections of the skin—herpes/warts (herpes) and furuncle/abscess (*furuncle*), urinary tract infections—bladder (*UTI*) and nephritis/pyelitis (*kidney*) were assessed as categorical data using the categories (c2–c7): none (c2); 1–2 times (c3); 3–4 times (c4); 5–6 times (c5); more than 6 times (c6); don’t know (c7). In section 4 of the ID Screen, antibiotic prescription (ABP) was assessed using the categories (c2–c6): none (c2); 1–3 times (c3); 4–6 times (c4); more than 6 times (c5) don’t know (c6). Missing values for both sections are depicted in category c1. For section 1 the full categorical range is used, exemplified by URTI, which show a good separation across all the categories, with 2 % of participants being placed in the highest category c6 “more than 6 times”. For antibiotic prescription, categories with a slightly different range were chosen, which are not fully exploited: 96 % of the data points are distributed across the categories none (c2) and 1–3 times (c3).

The 12-month cumulative incidence of certain infections (Section 1) as well as the frequency of antibiotic prescriptions over the past 12 months (Section 4) was measured in categories from “none” to “more than 6 times”. The distribution of the answers across the categories are shown in Figure 1. The item “Upper respiratory tract infection (URTI)” showed a full separation across the categories, with > 2 % of the participants being placed in the highest category “more than 6 times”. In contrast, the 12-month cumulative incidence for nephritis/pyelitis (kidney) is only represented by the categories “none” and “1–2 times”. Regarding antibiotic prescription (ABP), only 0.9 % and 0.7 % of the participants reported a frequency of prescription in the two highest categories “4–6 times” and “more than 6 times”, respectively.

The lifetime prevalence for the infectious diseases varied between 0 % (HIV) and 8 % (shingles) for the variables with a low frequency of missing values (< 5 %) across the 435 participants. Analysis stratified by study centers showed only for the frequency of chickenpox a significant variation. Participants in Bremen self-reported a significantly lower frequency for chickenpox than participants from Heidelberg (*p* = 0.025) and Freiburg (*p* = 0.003) (Tab. 2).

**Table 2:** Cumulative prevalence/lifetime prevalence of infectious diseases (Section 2 of the ID Screen) by study center

Total:	Bremen (HB)	Hamburg (HH)	Heidelberg (HD)	Freiburg (FR)	c^2^ test/Fisher’s exact test*
	*n* = 84	*n* = 91	*n* = 106	*n* = 156	
	%; [CI_95 %_]	%; [CI_95 %_]	%; [CI_95 %_]	%; [CI_95 %_]	
**Sepsis**	9.5; [4.9–17.7]	5.5; [2.4–12.2]	5.7; [2.6–11.8]	6.4; [3.5–11.4]	*p* = 0.910
**Sexually transmitted infections** (without HIV)	11.9; [6.6–20.5]	7.7; [3.8–15.09]	5.7; [2.6–11.8]	3.9; [1.8–8.1]	*p* = 0.390
**Infection of bones**	0	0	0.9; [0.2–5.2]	1.3; [0.4–4.6]	*p* = 0.980*
**Infection of joints**	3.6; [1.2–10.0]	6.6; [3.1–13.6]	7.6; [3.9–14.2]	5.8; [3.1–10.6]	*p* = 0.880
**Infection of the heart**	0	0	1,9; [0.5–6.6]	0	*p* = 0.650*
**Infection of the kidney**	9.5; [4.9–17.7]	4.4; [1.7–10.8]	3.8; [1.5–9.3]	5.1; [2.6–9.8]	*p* = 0.520
**HIV**	0	0	0	0	*p* = 1
**Chickenpox**	41.7; [31.7–52.3]**†	53.9; [43.7–63.7]	55.7; [46.2–64.8] †	59.6; [51.8–67.0]**	*p* = 0.027 **p = 0.003 (HB–FR) †*p* = 0.025 (HB–HD)
**Shingles**	2.4; [0.7–8.3]	11.0; [6.1–19.1]	10.4; [5.9–17.6]	9.0; [5.4–14.5]	*p* = 0.210

The differences between the study centers are not significant except for chickenpox

*Estimation with Fisher’s exact test for small cell numbers; **p-value between Bremen and Freiburg; †*p*-value between Bremen and Heidelberg

**Fig. 2:**
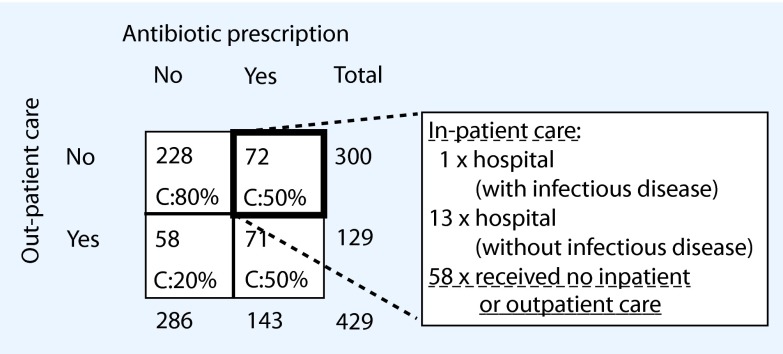
Antibiotic prescription in association with doctor’s visits. A total of 429/435 participants answered the question for antibiotics prescription (6 missing data): 143 (33 %) of the participants self-reported to have received antibiotic prescriptions in the past 12 months, out of which 72 (50 %) did not receive out-patient care. A subanalysis of these participants for in-patient care revealed that 14 received in-patient care and 58, equivalent to 41 % of participants, claiming to have received an antibiotic prescription, did not visit a physician in either in-patient or out-patient care

*HIV* human immunodeficiency virus,

Plotting antibiotic prescriptions against out-patient and in-patient care (Fig. 2) was used to appraise the validity of self-reported antibiotic prescriptions. A third of the participants, 143/431, stated to have been prescribed antibiotics, of whom 9 % (13/143) did not receive out-patient care but had been in the hospital for a non-infectious disease. Of all subjects who reported having been prescribed antibiotics, 41 % (58/143) did not visit a health care facility at all during the respective time period of 12 months.

Due to a duplicate request, a direct comparison of the answers to “12-month prevalence” of nephritis/pyelitis with reported “lifetime prevalence” of nephritis/pyelitis was possible and revealed that of the 5 participants who stated to have had a kidney infection in the past 12 months, 4 (80 %) claimed to never have had a kidney infection in their lifetime (diagnosed by a physician) or didn’t know if they had.

### Reliability

The reliability of the ID Screen could partly be assessed by comparing its results with similar questions from the core questionnaire. In the ID Screen (self-administered) and in the core questionnaire (CAPI) likewise, participants were asked if they ever had sepsis, chickenpox or shingles diagnosed by a physician. The agreement between these variables was 97.3 % for sepsis with a κ of 0.76 (CI_95 %_ 0.64–0.88), 70.5 % for chickenpox with κ = 0.46 (CI_95 %_ 0.40–0.53) and 93.2 % agreement for shingles with a κ  of 0.65 (CI_95 %_ 0.54–0.76).

### Construct validity

One aim of the ID Screen was to assess “susceptibility to infections”, using the seven items of section 1. The evaluation of the internal consistency resulted in a standardized Cronbach’s α of 0.36. One at a time removal of each item resulted in values in the range of 0.25–0.38 for this index. Removing the items “herpes”, “furuncle” and “kidney/pelvis”, respectively, led to an increase of Cronbach’s α to 0.37, 0.38 and 0.41.

**Fig. 3:**
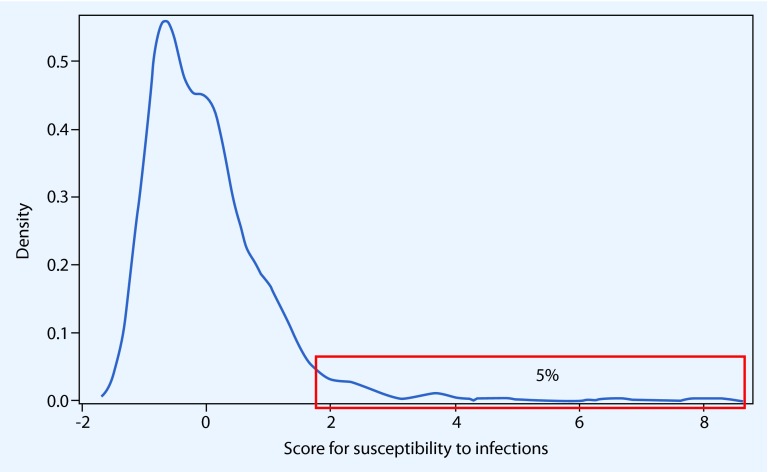
Distribution of the score assessing susceptibility to infections. Every subject with a factor value > 1.579 belongs to the group of the top 5 % and is considered to be highly susceptible for infections. This is the case for 21 subjects

EFA was used to evaluate the validity for the construct “susceptibility to infections” and to return a score. Due to missing data, 17 of the 435 subjects were omitted from the factor analysis. The correlation r was low, with |r| ranging between 0.012 and 0.242. Four factors were retained based on an Eigenvalue > 1. Application of the scree test extracted one meaningful factor, with an Eigenvalue of 1.6, explaining 23 % of the total variance. The KMO was 0.50 and omitting the variables abscess/furuncle and kidney infection due to an individual KMO < 0.5 would increase the overall KMO to 0.54. The density distribution of this factor is shown in Figure 3. The top 5 % of participants of the ID Screen had a score value > 1.6 and were considered highly susceptible for infections.

To evaluate if the ID Screen contributes to assessing the immune status, an EFA was performed with items judged to be relevant to influence and measure immune function (see methods).

The correlation ranged between |r| = 0.0001 and 0.65 and raw Cronbach’s α was 0.42. Removing the item “12-month prevalence of herpes/warts” would lead to an increase of Cronbach’s α to 0.45.

For the EFA 46 subjects were omitted from the analysis due to missing values. Nine factors were extracted based on an Eigenvalue > 1 and a scree test reduced the extracted factors to four, explaining 31 % of the total variance. The KMO was 0.60. The factor loadings above |0.4| for the included items are presented in Table 3.

**Table 3:** Factor loading for the construct “immune status”

Item	Factor 1 loading	Factor 2 loading	Factor 3 loading	Factor 4 loading
Allergies:				
Hay fever	0.64			
Bee venom				
Food	0.60			
Dust mites	0.65			
Animal hair	0.78			
Contact allergy				
Drug allergy		0.44		
Rheumatic diseases				
Autoimmune diseases				
Diseases of the skin				
Asthma	0.47			
Surgical removal of:				
Tonsils				0.61
Adenoid glands				0.48
Appendix				
Thymus			0.78	
Number of shingle infections			0.46	
Ever had an operation				0.60
12-month prevalence of infections:				
Upper respiratory tract infections		0.55		
Lower respiratory tract infections				
Gastro-intestinal tract infections		0.65		
Herpes				
Furuncle				0.43
Urinary tract infections		0.61		
Kidney/pelvis			0.76	

To calculate a score for “immune status”, 25 items were included in an EFA. Four factors were extracted following scree test criteria. Presented are only factor loadings > 0.40 or < − 0.40

Variables positively loading into the first factor were allergies and asthma. The second factor was a combination of drug allergy and the 12-month prevalence of upper respiratory tract, gastrointestinal tract and urinary tract infection. The variables loading into the third factor were removal of the thymus, shingles and 12-month prevalence of kidney infection. The fourth factor contains removal of tonsils and adenoid glands and surgeries in general.

## Discussion

We evaluated the ID Screen for its assessment of infections including resistance factors and its applicability as a self-report and self-administered tool to measure susceptibility to infections and immune status in a population-based study.

### Feasibility

With a participation rate between 87 % and 99 % and a range of 0.5–2 missing answers per questionnaire, the ID Screen turned out to be comprehensible and easy to use. Only one participant returned an incomplete form. However, comparing the results between the take-home and on-site approach returns a higher participations rate and shorter fill-in time for the on-site approach.

### Internal validity

An important aspect for the applicability of the ID Screen to assess susceptibility to infections and immune status is the full use of the applied categories for the respective items.

Both the sections 12-month prevalence of infections and prescription of antibiotics used six and five categories, respectively, from “none” to “more than 6 times” and “don’t know”. While 12-month prevalence of infections was covered adequately by these categories with URTI using all possible categories by at least 2 % of the participants, too many categories were applied to measure ABP (Fig. 1). The answer categories “4–6 times” and “more than 6 times” together were checked by only 1.6 % of the participants, thereby basically converting a categorized variable into a dichotomous variable (yes or no).

The direct comparison of the dual questions for an infection of the kidney or renal pelvis, once for 12-month prevalence and once for lifetime prevalence, revealed a lack of understanding by the subjects; four out of five subjects who reported an infection of the kidney or renal pelvis in the past 12-months did not report such an infection as lifetime infection diagnosed by a physician. We conclude that the question is asking for an answer a lay person does not know. The subjects do either not know what an infection of the kidney or renal pelvis is or had this infection not diagnosed by a physician.

Section 4 requests information about the prescription of antibiotics as a proxy for antibiotic use as an indicator for the severity of an infectious disease. However, this monitoring out of context does not allow an attribution for which infection the antibiotics were prescribed. Cross referencing antibiotic prescriptions with out-patient and in-patient care in the last 12 months as shown in Fig. 2, revealed a discrepancy: 41 % of the subjects who self-reported having received an antibiotic prescription, did not report any contact to a physician. An additional 9 % were in the hospital but unrelated to an infection according to self-disclosure. Several conclusions can be drawn from these results. One is that subjects might not understand the difference between prescription and intake of antibiotics, assuming that intake of antibiotics (e.g. left-overs from previous prescriptions) was counted as well. In addition, participants might not know in case of a hospitalization if they received antibiotics or not. The item assessing antibiotic prescriptions used examples for common antibiotics; however, the time frame referred to the past 12 months and subjects might not remember if or when they were prescribed antibiotics.

### Reliability

The most common method to test for reliability is the test–retest reliability. With the application of both the ID Screen and the core questionnaire of the GNC in Pretest 1, three questions were asked twice. For two variables Cohens’ κ was between 0.61 and 0.80 and the reliability therefore substantial. For the two items assessing chickenpox Cohen’s κ was moderate. The participants’ uncertainty about answering childhood diseases was also reflected in the high number of missing values for this item. An even greater degree of uncertainty could be observed for the assessment of vaccinations, which underscores the need to capture vaccination history on the basis of vaccination certificates (see Schultze et al. this issue).

Comparison of the acquired data for lifetime infections revealed differences among the study centers. For all except chickenpox the insignificant *p*-values allow the interpretation that the ID Screen is producing a robust response. For chickenpox these deviations confirm the uncertainty about answering childhood diseases.

### Construct validity

A very important aspect of a questionnaire is its ability to measure the intended constructs completely. For the ID Screen two of these constructs are “susceptibility to infections” and “immune status”.

The inter-item correlation for the seven variables “12-month prevalence of infections” is unacceptable with a Cronbach’s α of 0.36. This leads to the interpretation that these variables do not measure a latent construct, which results in a score for susceptibility to infections. This is confirmed by the factor analysis using the same variables. With a KMO of 0.50 the obtained score for susceptibility is barely acceptable for further analysis. The main factor loadings are the more common gastrointestinal infections, urinary tract infections and upper respiratory infections with kidney infections as the least frequent infection, loading the lowest. The factor can consequently be interpreted as frequency of infections instead of susceptibility to infection. A score, built over the sum of infections occurring in the past 12 months, has a similar result and the advantage that none of the subjects are omitted due to missing data (data not shown).

As different infections are caused by very different bacteria and viruses, which the immune system has different mechanism to fight against, it is not surprising that a factor comprising only the 12-month prevalence for 7 infections cannot adequately measure susceptibility.

The variables identified and expected to explain “immune status” within one score (see methods) may not fulfill this aim as apparent by the low inter-item correlation of 0.43. More variables might be needed to increase the intercorrelation, thereby describing a unidimensional construct. Clustering these variables in an EFA resulted in the extraction of four out of nine factors with an Eigenvalue > 1 using a scree test. The KMO is with 0.6 mediocre.

Factor loadings in Tab. 3 show that none of the factors solely explains immune status, and due to an unclear relationship between the items, factor naming is difficult. Factor 1 positively associates allergies with asthma and could be termed “allergies”. Factor 2 associates an allergy against drugs with the more common infections thereby describing “frequency of infections”. Factor 3 clusters the removal of the thymus with number of shingles infections and an infection of the kidney/pelvis. However both removal of the thymus and 12-month prevalence of infection of the kidney/pelvis have a very low frequency, which might explain their grouping in a factor “rare occurrences”. Factor 4 clearly associates the more common surgical removal of tonsils and adenoid glands with ever had an operation and represents “operations”.

Including dichotomous variables in a factor analysis is acceptable when they can be interpreted as a latent linear item, as is the case for having had an infection, no (0) or yes (1). However, including these variables leads to the extraction of more factors, which might explain the occurrence of nine factors with an Eigenvalue > 1. Nevertheless, the extracted four factors clearly do not explain the immune status, but solely aspects thereof. Especially the absence of diseases clearly associated with a defect in the immune response, rheumatic diseases, autoimmune diseases and diseases of the skin, like neurodermatitis, shows that with the current variables available, it is not possible to measure the “immune status”.

### Limitations

A typical validation study allows the measure of a new instrument against a gold standard. For a questionnaire assessing medical aspects, this can be the verification of answers against medical records, comparison with an already validated questionnaire or the measurement of suitable biomarkers. Due to the design of Pretest 1 as a feasibility study instead of a validation study, certain limitations like the impossibility of matching ID Screen answers with medical records were unavoidable. Furthermore, the infections assessed in section 1 are not typically associated with a doctor’s visit which would allow verification by medical records. To be able to control for information bias, a method allowing more frequent assessment of common infections (e.g. weekly) will be presented by Mall et al. in this issue.

To consider further aspects of external validity, like reliability and reproducibility, a reliability study was conducted during Pretest 2 (see Castell et al., this issue).

Due to possible selection bias and limited sample size, our results might be biased. Hence, the results might not be representative for different populations and should be interpreted with caution.

## Conclusion

The evaluation of the ID Screen revealed that this questionnaire is a suitable, short self-report tool when applied on-site in the study center and can measure infections; however, resistance factors like antibiotic prescriptions and hospitalization are not measured adequately.

One main concern is the complete assessment of risk factors and missing items to build a score for susceptibility and immune status. On the contrary, we identified unnecessary questions which could be removed from a revised version of the ID Screen in the benefit of time.

Therefore the following modifications and additions are recommended:

1. Removal of the question for 12 months or lifetime prevalence of kidney infections. The prevalence for both questions is very low, while the uncertainty and the chance of giving a false statement are very high.
2. Keeping the request for the lifetime prevalence for sepsis, STIs and HIV while removing the others. The above mentioned infections have either a good and reliable response rate or are indicators for the success of prevention measures or both. The items to be removed are difficult to understand, unreliable or too specific for a common questionnaire.
3. Pose the question for out-patient and in-patient care below the relevant 12-month prevalence items to allow identification of the corresponding infection and to exclude health care visits due to other diseases.
4. Remove the item for sick leave, as the subjects cannot distinguish between infectious and non-infectious causes.
5. Change the item for prescription of antibiotics. Use the categories from item 1 and ask for prescription by a physician, for present and past intake.
6. Simplify the request for the frequency of influenza vaccination. Possible answers could be: “on the average every year”, “about every other year”, “only one or two times”.
7. To measure immune status and susceptibility, more risk factors have to assessed, e.g. air-conditioning, hand hygiene, body hygiene, care of children, the sick and elderly.

## Electronic supplementary material

(DOCX 54 kb)

## Abbreviations

GNC: German National Cohort
KMO: Kaiser–Meyer–Olkin measure
EFA: Exploratory factor analysis
CAPI: Computer-assisted personal interviewing
URTI: Upper respiratory tract infections
ABP: Antibiotic prescription
