# Supplementary material for: Evaluation of a questionnaire to assess selected infectious diseases and their risk factors: Findings of a multicenter study
Source: Bundesgesundheitsblatt Gesundheitsforschung Gesundheitsschutz. 2014 Oct 10;57(11):1283–91. doi: 10.1007/s00103-014-2052-y (PMC4210746; doi:10.1007/s00103-014-2052-y)
Supplement: Supplementary file 1 — (DOCX 54 kb) [file 103_2014_2052_MOESM1_ESM.docx]

| ID-Number of participant: *<to be filled in by study personnel>* | | | | | | | |
| --- | --- | --- | --- | --- | --- | --- | --- |
| Beginning | | | | | | | |
| S1 | Date  (DD.MM.YYYY) | | | \|___\|___\| . \|___\|___\| . \|___\|___\|___\|___\|  D D M M YYYY | | | |
| S2 | Time  (h. min.) | | | \|___\|___\| . \|___\|___\|  h min | | | |
| Infectious diseases /infections The following section (questions IN1 to IN5) assesses the **frequency** of different infections in the past 12 month. Please sum up how often you suffered from these infections. If you had, for example, 3 times a cold and 1 time an infection of the middle ear, then you had 4 episodes of an infection (thus you check the answer option 3-4 times). If you had 7 times a cold and no other infection of the upper respiratory tract, then choose the answer option ‘more than 6 times’  **Important!** The questions IN1 to IN5 refer to the past 12 months. | | | | | | | |
| IN1 | How often have you had an infection of the upper respiratory tract (e.g. a cold, an infection of sinus, tonsils, middle ear, throat, larynx) in the past 12 months? | | | | | | |
|  | None | 1-2-times | 3-4-times | | 5-6-times | More than 6-times | Don’t know |
|  | 1 | 2 | 3 | | 4 | 5 | 6 |
| IN2 | How often have you had a bronchitis or pneumonia in the past 12 months? | | | | | | |
|  | None | 1-2-times | 3-4-times | | 5-6-times | More than 6-times | Don’t know |
|  | 1 | 2 | 3 | | 4 | 5 | 6 |
| IN3 | How often have you had an infection of the gastrointestinal tract (‘stomach flu’) in the past 12 months? | | | | | | |
|  | None | 1-2-times | 3-4-times | | 5-6-times | More than 6-times | Don’t know |
|  | 1 | 2 | 3 | | 4 | 5 | 6 |
| IN4 | How often have you had an infection of the skin or mucosa in the past 12 months?  a) Lip herpes, genital herpes, new warts | | | | | | |
|  | None | 1-2 times | 3-4 times | | 5-6 times | More than 6 times | Don’t know |
|  | 1 | 2 | 3 | | 4 | 5 | 6 |

This is a non validated translation of the ID screen (German – English). The original can be obtained upon request from the corresponding author

|  | How often have you had an infection of the skin or mucosa in the past 12 months?  b) Furuncle or abscess | | | | | |
| --- | --- | --- | --- | --- | --- | --- |
|  | None | 1-2 times | 3-4 times | 5-6 times | More than 6 times | Don’t know |
|  | 1 | 2 | 3 | 4 | 5 | 6 |
| IN5 | How often have you had a urinary tract infection in the past 12 months?  a) Urinary bladder (‘bladder infection‘) | | | | | |
|  | None | 1-2 times | 3-4 times | 5-6 times | More than 6 times | Don’t know |
|  | 1 | 2 | 3 | 4 | 5 | 6 |
|  | How often have you had a urinary tract infection in the past 12 month?  b) Kidney or renal pelvis | | | | | |
|  | None | 1-2 times | 3-4 times | 5-6 times | More than 6 times | Don’t know |
|  | 1 | 2 | 3 | 4 | 5 | 6 |

| **Important!** The following question (F1) refers to whether you **ever** had one of the following infections. | | | | | |
| --- | --- | --- | --- | --- | --- |
| F1 | Has a physician **ever** diagnosed one of the following infections? | | | | |
|  |  | Yes | No | Don’t know | No response |
|  | Blood poisoning (sepsis) | 1 | 2 | 3 | 4 |
|  | Acute, curable sexually transmitted infections (e.g. chlamydia, gonorrhea (‘clap’), syphilis) | 1 | 2 | 3 | 4 |
|  | Infection of a bone (osteomyelitis) | 1 | 2 | 3 | 4 |
|  | Infection of a joint | 1 | 2 | 3 | 4 |
|  | Infection of the heart valves (endocarditis) | 1 | 2 | 3 | 4 |
|  | Infection of a kidney or renal pelvis | 1 | 2 | 3 | 4 |
|  | HIV | 1 | 2 | 3 | 4 |
|  | Chickenpox | 1 | 2 | 3 | 4 |
|  | Shingles (herpes zoster) | 1 | 2 | 3 | 4 |
|  | If yes, how often have you had shingles? \|___\|___\| times | | | | |

| Hospital stay and medical treatment | | | | |
| --- | --- | --- | --- | --- |
| K1 | How often did you receive **outpatient** care (medical practice or clinic) in the past 12 months due to an **infectious disease**? | 1  2 | None  or  \|___\|___\| times |  |
| K2 | How often did you receive **inpatient** care in the past 12 months due to an **infectious disease** (i.e. you spent at least one night in the hospital)? | 1  2 | None  or  \|___\|___\| times |  |
| K3 | How many working days were you on sick leave in the past 12 months due to an **infectious disease**?  In case of several episodes, please sum up the days. | 1  2 | None  or  \|___\|___\|___\| days |  |
| K4 | Have you received outpatient care in a hospital in the past 12 months **for another reason** (i.e. not because of an infectious disease)? | 1  2  3 | Yes  No  Don’t know |  |
| K5 | Have you received inpatient care in a hospital in the past 12 months **for another reason** (i.e. not because of an infectious disease)? (i.e. you spent at least one night in the hospital)? | 1  2  3 | Yes  No ------------🡪 continue with question  Don’t know -------------🡪 continue with question | **K6**  **K6** |
| K5a | If yes, how long?  If you have received inpatient care several times, please sum up the episodes. |  | \|___\|___\|___\| nights |  |
| K5b | If yes, in which unit?  **Multiple answers possible** | 1  2  3  4 | Internal Medicine  Surgery  Intensive care unit  Other, which…………………………………  …………………………………………………… |  |
| K6 | Have you **ever** undergone surgery? | 1  2  3 | Yes  No ------------🡪 continue with question  Don’t know ------------🡪 continue with question | **M1**  **M1** |
| K6a | Have you had surgery in the past 12 months | 1  2  3 | Yes  No  Don’t know |  |
| K7 | Have you **ever** undergone the following surgical procedures?  **Multiple answers possible** | 1  2  3  4  5 | Removal of the pharyngeal and palatal tonsils  Removal of the polyps of the paranasal sinuses  Removal of the appendix  Removal of the spleen  Removal of the thymus | Year  \|__\|__\|__\|__\|  \|__\|__\|__\|__\|  \|__\|__\|__\|__\|  \|__\|__\|__\|__\|  \|__\|__\|__\|__\| |

| Medicines | | | | |
| --- | --- | --- | --- | --- |
| M1 | How often did a physician prescribe antibiotics (drugs against infections; e.g. Penicillin, Augmentan, Tavanic; but no ointments for external use) in the past 12 months?  **Include as well if you did not take the medicine!** | 1  2  3  4  5 | None  1-3-times  4-6-times  More than 6 times  Don’t know |  |
| M1a | How certain are you relating your statement (Question M1)? |  | Very certain  Rather certain  Neither nor  Rather uncertain  Very uncertain |  |

| Vaccination | | | | |
| --- | --- | --- | --- | --- |
| V1 | Have you **ever** been vaccinated against the flu (influenza)? | 1  2  3 | Yes  No ------------🡪 continue with question  Don’t know ------------🡪 continue with question | **V2**  **V2** |
| V1a | If yes, how often do you get vaccinated against the flu (influenza)? | 1  2  3  4 | Every year  In average every second year  In average every third year or less  Don’t know |  |
| V1b | When were you vaccinated for **the first time** against the flu (influenza)? | 2 | Year: \|___\|___\|___\|___\|  or  Don’t know |  |
| V1c | When were you vaccinated for **the last time** against the flu (influenza)? | 2 | Year: \|___\|___\|___\|___\|  or  Don’t know |  |
| V2 | Have you **ever** been vaccinated (e.g. ‘Pneumovax 23’) against pneumococci (pathogen causing pneumonia)? | 1  2  3 | Yes  No ------------🡪 continue with question  Don’t know ------------🡪 continue with question | **T1**  **T1** |
| V2a | If yes, when were you vaccinated? | 2 | Year: \|___\|___\|___\|___\|  or  Don’t know |  |

| Animals | | | | | | | | | | |
| --- | --- | --- | --- | --- | --- | --- | --- | --- | --- | --- |
| T1 | Have you **ever** had regular contact to pets on a private or professional basis over a period of more than six months? | 1  2  3 | Yes  No -------🡪 continue with question  Don’t know -------🡪 continue with question | | | | | | | **T2**  **T2** |
|  | If yes, with which animal/s? | | | | | | | | | |
|  |  | | | Yes | | No | | Don’t know | | |
|  | Dog | | | 1 | | 2 | | 3 | | |
|  | Cat | | | 1 | | 2 | | 3 | | |
|  | Rodent (hamster, rabbit, guinea pig) | | | 1 | | 2 | | 3 | | |
|  | Caged bird | | | 1 | | 2 | | 3 | | |
|  | Reptiles | | | 1 | | 2 | | 3 | | |
|  | Fish | | | 1 | | 2 | | 3 | | |
|  | Other pets, if yes, which? | | | ………………………………………….. | | | | | | |
| T2 | Do pets live in your household? | 1  2  3 | Yes  No -------🡪 continue with question  Don’t know -------🡪 continue with question | | | | | | | **T3**  **T3** |
|  | If yes, which pets? | | | | | | | | | |
|  |  | | | Yes | If yes, number of | | No | | | Don’t know |
|  | Dog | | | 1 | ………... | | 2 | | | 3 |
|  | Cat | | | 1 | ………... | | 2 | | | 3 |
|  | Rodent (hamster, rabbit, guinea pig) | | | 1 | ………... | | 2 | | | 3 |
|  | caged bird | | | 1 | ………... | | 2 | | | 3 |
|  | Reptiles | | | 1 | ………... | | 2 | | | 3 |
|  | Fish | | | 1 | ………... | | 2 | | | 3 |
|  | Other pets, if yes, which? | | | ………………………………………….. | | | | | | |
| T3 | Do you or someone else living in your household have contact to livestock on a **professional basis**, e.g. farmer or veterinarian, etc.? | 1  2  3 | | Yes, myself  Yes, someone else living in my household  No 4 Don’t know | | | | | | |
| End of survey | | | | | | | | | | |
| S3 | Time end of survey  (h. min.) |  | | \|___\|___\| . \|___\|___\|  h min. | | | | |  | |

| Kommentare | | |
| --- | --- | --- |
| C1 | In case you have comments or questions concerning the questionnaire, please let us know. Also, you can report here any other problems (problems of comprehension etc.) that you encountered during completion of the questionnaire.  ………………………………………………………………………………………………………………….  ………………………………………………………………………………………………………………….  ………………………………………………………………………………………………………………….  ………………………………………………………………………………………………………………….  ………………………………………………………………………………………………………………….  ………………………………………………………………………………………………………………….  ………………………………………………………………………………………………………………….  ………………………………………………………………………………………………………………….  ………………………………………………………………………………………………………………….  …………………………………………………………………………………………………………………. |  |

**Thank you so much for your participation!**
